# Exploring the potential of tocols and retinoids in authenticating pasture-derived sheep milk and cheese

**DOI:** 10.3168/jdsc.2025-0982

**Published:** 2026-03-12

**Authors:** Nagore Iglesias-Rodrigo, María Ángeles Bustamante, Noelia Aldai, Paz Lavín, Teresa Manso, Ángel R. Mantecón, Luis Javier R. Barron

**Affiliations:** 1 Lactiker Research Group, Department of Pharmacy and Food Sciences, University of the Basque Country (EHU), 01006, Vitoria-Gasteiz, Spain; 2 Higher Technical School of Agricultural Engineering, University of Valladolid (UVa), 34004, Palencia, Spain; 3 Mountain Livestock Institute, Spanish National Research Council (CSIC)–University of León (ULE), 24346, Vega de Infanzones, León, Spain

## Abstract

This study investigated the potential of tocols and retinoids to differentiate sheep milk and cheese from concentrate-feeding indoor farms and pasture-feeding extensive farms. A total of 94 milk and 90 cheese samples collected from intensive and extensive commercial farms were analyzed using high-performance liquid chromatography to quantify α- and γ-tocopherol, α- and β-tocotrienol, retinyl palmitate, and retinol. Higher concentrations of tocols and retinoids were found in the samples from extensive compared with intensive farms. The results of discriminant analysis showed that tocols and retinoids were able to clearly distinguish between the 2 farming systems. In this regard, a new multiple marker named vitamin-active lipid compounds indicator showed a strong discriminative power between intensive and extensive farms (91.4% correct classification for milk and 100% for cheese samples). These findings confirm that this indicator is a reliable tracer to authenticate dairy products according to production system, of particular interest for pasture-based foods.

## Highlights

• Pasture-based farms show higher contents of VALC.
• Tocols and retinoids clearly differentiate sheep milk and cheese by farming system.
• A new multiple indicator was generated to authenticate pasture-derived dairy foods.
• The multiple indicator was based on a ratio between tocol and retinoid molar contents.

## Graphical Abstract

This study evaluated the potential of vitamin-active lipid compounds (VALC) specifically retinoids and tocols (a-tocopherol, a-tocotrienol, and γ-tocopherol) as biochemical tracers to authenticate pasture-derived sheep milk and cheese. By comparing intensive indoor concentrate feeding with extensive grazing, the VALC indicator effectively reflected the dietary transfer of lipids into dairy products. Milk and cheese from grazing based farms showed higher retinoid and a-tocopherol contents, and, in consequence, higher values of the VALC indicator. Discriminant analysis demonstrated strong classification performance, correctly identifying dairy products from extensive farms with 100% accuracy for milk and 91% for cheese. These results highlight the robustness of the VALC indicator as an analytical tool for distinguishing commercial dairy products based on farming system, supporting food authenticity within the sheep dairy sector.

**Figure:**
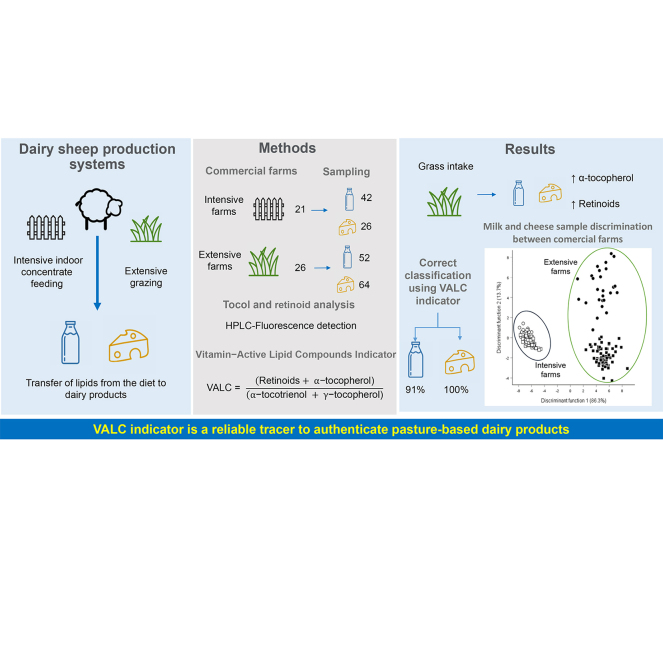

Small ruminant production systems involve important socioeconomic and environmental aspects in developing regions or in marginal areas of developed regions (de Rancourt et al., 2006; Barron et al., 2021). Most non-cow dairy ruminant species are adapted to harsh environments and to grazing in marginal areas that are, in general, locations of low economic profitability but which possess high natural and cultural value (Lomba et al., 2017; Garmendia et al., 2022). Despite limitations due to the small-scale operations, small ruminants contribute to the production of traditional and high added-value dairy products that are associated with regional or local origin. Consumption of ewes' milk is low but is intended for the production of fermented foods such as cheese and, consequently, milk composition is directly related to the production of high-quality dairy products (Barron et al., 2018).

The quality of raw milk is dependent on factors linked to animal management, such as genetic, physiological, or dietary factors (Lucas et al., 2006b). These factors are particularly relevant in the case of high-quality dairy products, greatly valued by consumers, and which may be protected by quality labels such as Protected Designation of Origin or Protected Geographical Indication in Europe, closely linked to milk production conditions (Barron et al., 2018). Among these conditions, pasture-based diets are particularly noteworthy, as they strengthen the connection between dairy products and their origin; in addition, as grazing systems are often positively perceived by consumers, and composition of fresh forage can confer specific compositional and sensory attributes to dairy product. At present, there is growing interest in animal food products obtained from free-grazing animals, perceived by many consumers as high-quality foods with positive ethical notes related to animal welfare and environmental benefits (Etaio et al., 2025).

As consumer attitudes increasingly focus on production practices, the authentication of foods derived from different livestock systems, particularly grazing systems, has become an important area of research. The authentication of dairy foods obtained under grazing management can rely on the identification of specific compounds transferred directly from fresh forage to milk, as well as metabolites produced by ruminal microbiota or by the animal's metabolism in response to a grass-based diet (Capuano et al., 2014). Previous studies have shown that milk and dairy products from ruminants raised on pasture-based farms exhibit a distinct nutrient profile compared with products obtained from animals fed total mixed rations (Moscovici Joubran et al., 2021). Although the scientific literature is not very exhaustive, evidence indicates that, regardless of the botanical diversity of the pastures, the consumption of fresh grass can confer unique nutritional and sensory characteristics to milk, compared with diets based on conserved forages or cereal-based concentrates (Coppa et al., 2019; Cifuni et al., 2022). Consequently, milks and dairy foods from grazing animals contain significant amounts of various bioactive compounds that contribute to beneficial effects on human health (Martini et al., 2021; Moscovici Joubran et al., 2021).

It is well established that animal feeding is one of the main factors influencing the physicochemical composition and nutritional quality of milk, particularly its lipid profile, including fatty acid composition and other minor compounds such as tocopherols, retinoids, and cholesterol (Nájera et al., 2017). In this context, several lipid compounds have been proposed as potential tracers of grazing-based dairy products (Morand-Fehr et al., 2007; Gutiérrez-Peña et al., 2018; Garmendia et al., 2022). To date, the principal lipid tracers suggested for authenticating pasture-based foods include fatty acids, tocopherols, and carotenoids originating from the animals' diet, as well as compounds formed through ruminal biohydrogenation or enzymatic conversion in the intestinal mucosa (Aldai et al., 2013; Capuano et al., 2014). However, the limited number of studies, particularly those conducted under commercial farm conditions, and the considerable variability reported across research due to differences in species, geographic regions, pasture types, and environmental or grazing conditions make the authentication of pasture-based dairy products a significant challenge for practical food control.

With regard to liposoluble compounds with vitamin activity, sheep dairy products are an excellent source of vitamins A and E (Raynal-Ljutovac et al., 2008). Vitamin A is an isoprenoid-derived compound (Cabezuelo et al., 2020) that occurs in sheep milk as retinoids. Sheep milk fat accumulates retinol in esterified form, mostly as retinyl palmitate (Balthazar et al., 2017), as dietary β-carotene is enzymatically converted to retinol in the intestinal epithelium by β-carotene monooxygenase during absorption through the intestine wall (Álvarez et al., 2014; Bernard et al., 2018). Studies on retinoids in milk consistently report higher contents in milk fat from grazing animals compared with animals fed conserved forages and concentrate-based diets (Hulshof et al., 2006; Delgado-Pertíñez et al., 2013). This is largely explained by the fact that the plant species constituting fresh pasture contain substantial amounts of β-carotene (Valdivielso et al., 2016), and that β-carotene levels are typically higher in fresh grass than in conserved forages or standard commercial concentrates (Nozière et al., 2006; Morand-Fehr et al., 2007). Vitamin E comprises 8 naturally occurring isoforms—4 tocopherols and 4 corresponding tocotrienols—characterized by a chromanol ring and phytyl side chain, and designated as α-, β-, γ-, and δ-isomers according to the number and position of methyl group substituents. Among these, α-tocopherol exhibits the highest biological activity. Similar to retinoids, tocopherol concentrations in sheep milk fat are usually higher under grazing management than under indoor feeding systems (Valdivielso et al., 2015). Fresh pasture species are known to be rich in tocopherols, particularly α-tocopherol, which can be efficiently transferred to milk fat (Pizzoferrato et al., 2007; Valdivielso et al., 2016). Moreover, fresh grass generally contains greater amounts of tocopherols than conserved forages and standard commercial concentrates do (Morand-Fehr et al., 2007).

In this context, the aim of this study is to differentiate milk and cheese produced on concentrate-based indoor farms and pasture-based extensive farms based on their tocopherol and retinoid compositions, and to propose a multiple vitamin-active lipid compounds (**VALC**) indicator that may facilitate the authentication of milk and cheese from pasture-based systems. The study focused on the 2 main management systems that characterize the sheep dairy sector in southern Europe, particularly in Spain: intensive production in the Castile and León region, and extensive grazing systems in the Basque Country and Navarre. The latter area represents the Atlantic European temperate marginal lands, with a long tradition of sheep herding and production of high-value sheep dairy products (Andonegi et al., 2021; Garmendia et al., 2022). In contrast, Castile and León exemplifies a European region that has undergone a large-scale transition from grazing-based to indoor-feeding intensive dairy sheep production. This study adopts a holistic perspective and is grounded in the hypothesis that, despite variability related to factors such as breed, farm size and geographical location, environmental conditions, and specific management practices, the fundamental distinction between grazing and concentrate-plus-forage feeding should be sufficient to discriminate milk and cheese from extensive versus intensive commercial farms.

The study included 21 intensive commercial farms from Castile and León and 26 extensive commercial farms from the Basque Country and Navarre. Intensive farms are characterized by year-round stabling of the specialized Assaf breed, concentrate- and forage-based feeding without access to pasture, continuous lambing, and annual milk production. In contrast, extensive farms raise the autochthonous Latxa breed under grazing conditions, with single annual lambing and milking season lasting 6 to 7 mo, and with short periods of stabling depending on pasture availability. Seasonal milk production typically begins in January. During the first 3 mo, farms follow a semi-extensive management system characterized by indoor forage and concentrate feeding, complemented by part-time grazing depending on pasture availability. Grazing intensity progressively increases, and, from April onward, animals are exclusively grass-fed outdoors. Bulk raw milk samples (1 L) were randomly collected in duplicate from all farms. Samples from intensive farms were collected between January and December, covering a sampling window of 5 to 10 mo, as these farms produce bulk milk continuously with a relatively constant fat-to-protein ratio throughout the year. Samples from extensive farms were collected from late May to mid-June, with a 2-wk interval between replicates, corresponding to the period when flocks were managed outdoors under high-intensity grazing. In total, 42 and 52 bulk milk samples were collected from intensive and extensive commercial farms, respectively. All milk samples were transported to the laboratory under cooling conditions, subsampled, and stored at −80°C until analysis. Whole cheese samples were also randomly collected from a subset of the farms participating in the milk sampling. In total, cheeses were obtained from 16 extensive and 3 intensive farms. The lower number of intensive farms producing cheese reflects the fact that most dairy sheep farms in Castile and León sell milk to the dairy industry rather than processing it on-farm. Both farm types used bulk raw milk to produce semi-hard cheeses: Zamorano-type in intensive farms and Idiazabal-type in extensive farms. All cheeses weighed between 1 and 1.5 kg and were aged for approximately 5 mo. Cheese production coincided with the milk sampling week, and 2 cheeses were collected from each weekly batch (4 cheeses per farm). To increase the number of Zamorano-type cheeses, 2 additional cheeses were collected from each intensive farm during the milk sampling period, and other 4 cheeses were obtained from 2 intensive dairies not included in the milk sampling. In total, 26 and 64 cheeses were collected from intensive and extensive commercial farms, respectively. All cheeses were transported to the laboratory under cooling conditions, subsampled, vacuum-sealed, and stored at −40°C until analysis. Milk and cheese samples were analyzed in separate batches and in random order, regardless of farm and sampling date.

Tocols (tocopherols and tocotrienols) and retinoids were simultaneously extracted from 1 mL of liquid milk previously thawed in a water bath at 25°C for 60 min, using 2 liquid-liquid extraction steps without saponification, following the method described by Valdivielso et al. (2015). For cheese samples, tocols and retinoids were extracted from 500 mg of grated cheese by solid-liquid extraction followed by saponification, as previously described (Panfili et al., 1994; Valdivielso et al., 2015). Tocols and retinoids where simultaneously analyzed using normal-phase HPLC (model 1260, Agilent Technologies, Madrid, Spain) coupled to a fluorescence detector (1260 FLD Spectra, Agilent Technologies) as previously described (Blanco-Doval et al., 2025). Briefly, the compounds were separated on a column 10 cm long, with 3-mm i.d. and 3-µm particle size (Luna Silica, Phenomenex, Madrid, Spain) using a 15-min nonlinear gradient elution of 1,4-diohexane/n-hexane (Merck, Darmstadt, Germany) and a flow rate of 1 mL/min. The fluorescence detector operated at 298 nm excitation, with emission wavelength of 328 nm for tocols and 475 nm for retinoids. Peaks were identified by comparing the retention times of sample compounds with those of high-purity commercial standards. Quantification was performed by external calibration using standard solutions of α-, β-, γ-, and δ-tocopherol, α-tocopherol acetate, α-, β-, γ-, and δ-tocotrienol, retinol, retinyl acetate, retinyl propionate, and retinyl palmitate (>99% purity, Merck) in n-hexane/isopropanol 99/1 (vol/vol; Merck). Standards were analyzed in triplicate in 4 concentration levels from 0.01 to 0.5 µg/mL. Linearity was verified, and coefficient of determination (R^2^) exceeded 0.995 for all analytes. Relative standard deviations (**RSD**) for 5 replicates at the lowest and highest concentrations were below 7%, and the recovery values for all tocols and retinoids ranged from 88% to 111%. The limit of quantification (**LOQ**) values were determined by analyzing 10 replicates of the lowest standard concentration giving RSD below 20%. This concentration corresponded to an LOQ of 1.5 µg/100 g for all tocols and retinoids in milk and cheese matrices. Final concentrations were expressed in micrograms (µg) per 100 g, using 3 significant figures.

The multiple VALC indicator was calculated according to Equation [1]. The VALC indicator values are dimensionless and were calculated for both milk and cheese samples using the molar content (µmol/100 g) of tocols and retinoids. Molar contents were used to calculate the indicator because, as indicated earlier, the absence of retinyl palmitate in cheese was a direct consequence of the saponification reaction occurring during the extraction process. As this reaction is governed by molar ratios, the indicator was therefore calculated on a molar basis:

[1] $$\mathrm{VALC}=\frac{\left(\mathrm{retinoids}+\alpha -\mathrm{tocopherol}\right)}{\left(\alpha -\mathrm{tocotrienol}+\gamma -\mathrm{tocopherol}\right)}.$$

The rationale behind the VALC indicator is that higher values reflect a greater molar content of lipid compounds with vitamin activity. Accordingly, the numerator includes the molar content of retinoids (vitamin A) and α-tocopherol, the tocol isomer with the highest vitamin E activity, whereas the denominator includes the molar content of tocol isomers with lower vitamin activity (Debier et al., 2005; Martini et al., 2021).

IBM SPSS Statistics version 28 (IBM Corp., Armonk, NY) was used for statistical analyses. Only those individual compounds present above the LOQ in at least 60% of both intensive and extensive milk and cheese samples were included in the statistical processing. For cheeses, compounds also had to exceed the LOQ in at least half of the cheeses collected from each farm. Tocol and retinoid contents were log-transformed, and data were checked for normality, homoscedasticity, and residual randomness. A general linear model (ANOVA) was applied, considering management system (intensive and extensive management) as fixed factor and individual commercial farm as random factor nested within management system. A stepwise linear discriminant analysis (**DA**) was then performed on the combined data set, using individual tocol and retinoid contents to discriminate between intensive and extensive commercial farms. Wilks' λ statistic was used to assess the differences in the discriminant variables between groups. Additionally, separate DA were conducted for milk and cheese samples using the VALC indicator to assess its ability to classify samples according to farm management system. For milk, equal prior probabilities were assumed for the 2 farm types, whereas for cheese, due to unequal sample sizes and differing data dispersion, prior probabilities were calculated for each group. For both milk and cheese samples, the within-group covariance matrix was used. Classification results were submitted to cross-validation. In both milk and cheese sample cases, the aim of DA was to optimize the overall classification accuracy. Statistical significance was declared at *P* ≤ 0.05.

Table 1 shows the content of tocols and retinoids in sheep milk and cheese samples from indoor feeding intensive and pasture-based extensive commercial farms. As observed, the total content of tocols was higher than that of retinoids in milk and cheese samples for both intensive and extensive farms, as other authors have reported for sheep milk (Valdivielso et al., 2016). Regarding retinoids, retinyl palmitate was the only esterified compound found in all milk samples, whereas other esterified retinoids such as acetate and propionate were detected in very few milk samples. Previous studies have indicated that retinoids in foods from animal origin occur mainly as retinyl esters of long-chain fatty acids, and preferentially esterified with palmitic acid (Rocchi et al., 2016). However, as expected, retinol was the only retinoid compound found in cheese samples, mostly due to the saponification reaction used for the extraction procedure. As mentioned before, previous studies have reported that farming practices, in particular those related to feeding management, are important factors affecting the content of vitamin A in milk and cheese. Concerning this, the contents of retinyl palmitate in milk and of retinol in cheese samples were significantly higher (*P* ≤ 0.001) in pasture-based extensive than in indoor-feeding intensive commercial farms. These results are consistent with those obtained in other studies, in which the retinoid content was higher in milk from animals fed on pasture compared with indoor feeding (Fedele et al., 2004; Lucas et al., 2006a). The highest amount of retinol in extensive cheeses also agrees with other studies, which reported that the effect of the animals' diet was predominant compared with the technological conditions used for cheesemaking (Lucas et al., 2006b; Fantuz et al., 2016). However, it is also interesting to remark that the content of vitamin A in form of retinoids, or its precursor β-carotene, in milk and cheese from other animal species is also higher when animals are under grazing management compared with indoor feeding based on ensiling or drying forages and concentrates (Nozière et al., 2006; O'Callaghan et al., 2017).

**Table 1:** Mean content (μg/100 g) and standard deviation of fat-soluble vitamin compounds in sheep milks and cheeses from intensive and extensive commercial farms and statistical significance (*P*-value) for the management system effect^1^

Item	Milk			Cheese		
	Intensive farms	Extensive farms	*P*-value	Intensive farms	Extensive farms	*P*-value
Retinyl palmitate	87.5 ± 20.3	127 ± 26	≤0.001	<LOQ	<LOQ	—
Retinol	<LOQ	<LOQ	—	140 ± 33	246 ± 32	≤0.001
α-Tocopherol	109 ± 37	169 ± 59	≤0.001	387 ± 91	636 ± 22	≤0.001
γ-Tocopherol	9.60 ± 2.36	5.83 ± 1.40	≤0.001	59.3 ± 17.9	23.3 ± 5.8	≤0.001
α-Tocotrienol	7.80 ± 2.19	5.93 ± 1.66	≤0.001	47.5 ± 9.9	22.5 ± 6.7	≤0.001
β-Tocotrienol	6.73 ± 2.19	8.06 ± 2.20	0.062	21.8 ± 8.4	28.5 ± 1.7	0.166

1 β- and δ-Tocopherol, γ- and δ-tocotrienol, α-tocopherol acetate, retinyl acetate, and retinyl propionate were found in very few milk or cheese samples. LOQ = limit of quantification.

Regarding tocols, α-tocopherol was the most abundant isomer, followed at a considerable distance by γ-tocopherol and α- and β-tocotrienol in both milk and in cheese samples from both intensive and extensive farms (Table 1). This distribution aligns with previous finding in milk from various animal species (Valdivielso et al., 2015; Nájera et al., 2017; Gutiérrez-Peña et al., 2018; Blanco-Doval et al., 2025). Other tocols, including δ-tocopherol, δ-tocotrienol, and α-tocopherol acetate, were not detected in any samples, whereas β-tocopherol and β-tocotrienol appeared in very few milk and cheese samples. Comparing production systems, α-tocopherol contents were significantly higher (*P* ≤ 0.001) in both milk and cheese from pasture-based extensive farms compared with indoor-feeding intensive farms. On the contrary, γ-tocopherol and α-tocotrienol contents were significantly higher (*P* ≤ 0.001) in milk and cheese from intensive farms, whereas β-tocotrienol did not differ significantly between systems (*P* > 0.05). As with retinoids, the animals' diet plays a decisive role in shaping tocol level in dairy products. The highest tocol content, particularly α-tocopherol, in samples from pasture-based extensive farms agrees with previous studies reporting higher α-tocopherol content in milk from pasture-fed animals compared with those kept indoors (Agabriel et al., 2007; Valdivielso et al., 2015; Marino and Schadt, 2016).

Taking into account the differences observed in the individual contents of retinoids and tocols in milk and cheese from indoor-feeding intensive and pasture-based extensive commercial farms, a stepwise linear DA was applied to evaluate, from a multivariate perspective, the ability of these compounds to distinguish dairy samples between production systems. Figure 1 displays the distribution of samples along the 2 canonical discriminant functions. As shown, milk and cheese samples were clearly separated according to their origin (intensive vs. extensive). A greater dispersion was observed among samples from extensive farms, along with a more marked differentiation between milk and cheese within this group. This likely reflects the greater variability in grazing conditions and animal management among extensive farms compared with the more standardized feeding and management practices characteristic of intensive farms. Additionally, the use of standardized milk in cheesemaking on intensive farms contributed to the lower dispersion observed in cheese samples, in contrast to the greater variability associated with the use of nonstandardized milk in extensive farms.

**Figure 1:**
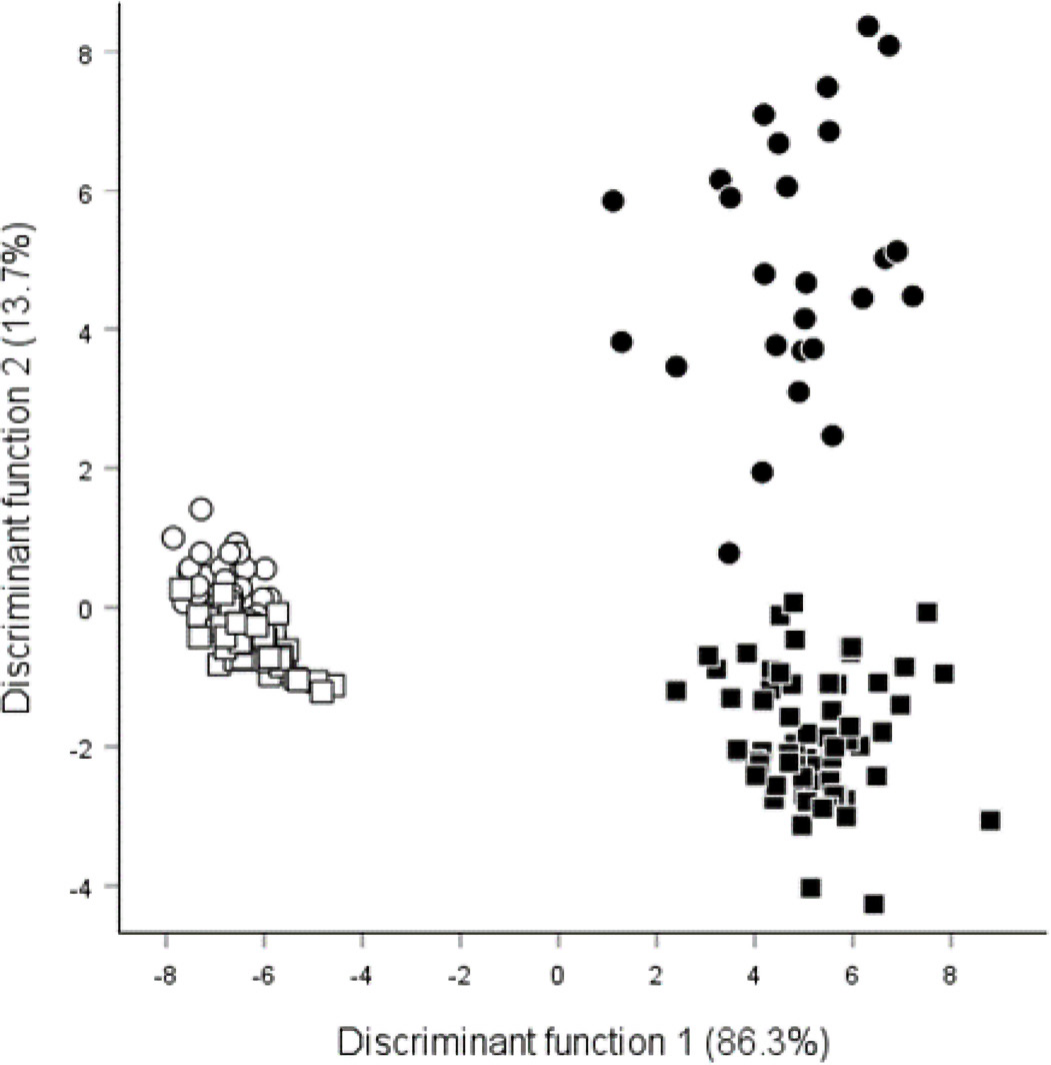
Graph for the 2 canonical discriminant functions corresponding to the stepwise linear discriminant analysis on fat-soluble vitamin compounds of sheep milk and cheese samples from the intensive and extensive commercial farms. ○ = milk from intensive farm; □ = cheese from intensive farm; ● = milk from extensive farm; ■ = cheese from extensive farm.

In advancing the authentication of dairy foods derived from pasture-based systems, one key aspect is the development of robust indicators capable of establishing product authenticity or revealing the presence of fresh grass-related compounds (Garmendia et al., 2022). These indicators must also be quantitatively measurable in the final product, thereby linking the product to specific production characteristics (Barron et al., 2018). In line with the results mentioned herein, increasing VALC indicator values should be strongly associated with higher content of fat-soluble vitamins in milk and cheese. Table 2 shows mean values, SD, and upper and lower limits of the 95% CI for the VALC indicator in milk and cheese from indoor-feeding intensive and pasture-based extensive commercial farms. As expected, mean values were significantly higher (*P* ≤ 0.001) for both matrices from extensive farms. Notably, mean VALC values were approximately twice as high in milk and 5 times higher in cheese from extensive farms. These marked differences, together with the narrow confidence intervals within each sample and farm type, highlight the strong potential of VALC as an indicator for authenticating dairy products in relation to grazing management. However, according to the definition of the VALC indicator (Equation [1]), it is worth noting that the higher content of individual tocols with lower vitamin activity (γ-tocopherol and α-tocotrienol) in milk and cheese from intensive farms (Table 1) contributed to reducing VALC indicator values in these farms, thereby increasing the differences versus extensive farms. This fact could suggest a coincidence between the discriminatory potential of the indicator and the biological activity of the compounds; however, this situation should not be generalized, because the content of these minor compounds in milk and cheese could vary considerably under other intensive conditions. In the scientific literature, a related metric has been proposed, referred to as an antioxidant capacity indicator and defined as the ratio between the total contents of tocols and terpenoids and the content of retinoids. This indicator showed significant and progressive increases in sheep milk and cheese from winter indoor feeding to mid-spring part-time grazing, and finally to extensive mountain grazing in late spring (Garmendia et al., 2022). In the same way, although the present study did not include milk and cheese samples from part-time grazing, it can be anticipated that VALC values would progressively increase from indoor feeding to high-intensity grazing, with part-time grazing representing an intermediate stage. Finally, the authentication capacity of the VALC indicator was assessed through a DA applied separately to milk and cheese samples, with the aim of classifying them according to their farm of origin. The analysis showed that 100% of cheese samples were correctly assigned to their corresponding group. For milk, 38 of 42 samples from intensive farms and 48 of 52 from extensive farms were correctly classified, yielding an overall accuracy of 91.4%. Cross-validation confirmed the results, yielding the same classification percentages for both milk and cheese samples.

**Table 2:** Mean values, standard deviation, and upper and lower limits of the 95% confidence interval for the vitamin-active lipid compounds (VALC) indicator in sheep milk and cheese from intensive and extensive commercial farms

Item	Milk		Cheese	
	Intensive farms	Extensive farms	Intensive farms	Extensive farms
Mean	10.4^b^	22.9^a^	5.71^b^	22.5^a^
SD	3.2	6.8	1.71	7.1
Lower limit	9.38	21.0	5.02	20.8
Upper limit	11.4	24.8	6.40	24.3

a,b Means with different superscript letters are significantly (*P* ≤ 0.001) different between intensive and extensive farms for milk and cheese samples.

In conclusion, the results reported in this study indicate that sheep milk and cheese produced under grazing conditions accumulate higher levels of VALC, such as tocols and retinoids, than products from animals managed under indoor-feeding intensive systems. Moreover, differences in the contents of these compounds could distinguish milk and cheese from indoor-feeding intensive and pasture-based extensive sheep farming systems under a holistic framework; that is, independent of breed, geographical location, indoor feeding conditions, grazing management practices, or environmental conditions. The multiple VALC indicator showed strong potential for authenticating dairy products according to production systems, which is particularly valuable for products derived from grazing systems, as highlighted earlier. Nevertheless, further research encompassing a wider range of production contexts and a substantially larger sample size is needed to ensure the robustness and reliability of the VALC indicator in authenticating pasture-based dairy products. In addition, the implementation of food authentication indicators such as the one proposed in this study will require context-specific adaptation and thorough validation to guarantee their applicability across diverse settings. Ultimately, the authentication of pasture-based products represents a key strategy for supporting and promoting grazing systems, which are currently at risk of abandonment in many regions worldwide.

## References

[bib1] Agabriel C., Cornu A., Journal C., Sibra C., Grolier P., Martin B. (2007). Tanker milk variability according to farm feeding practices: Vitamins A and E, carotenoids, color, and terpenoids. J. Dairy Sci..

[bib2] Aldai N., De Renobales M., Barron L.J.R., Kramer J.K.G. (2013). What are the *trans* fatty acids issues in foods after discontinuation of industrially produced *trans* fats? Ruminant products, vegetable oils, and synthetic supplements. Eur. J. Lipid Sci. Technol..

[bib3] Álvarez R., Meléndez-Martínez A.J., Vicario I.M., Alcalde M.J. (2014). Effect of pasture and concentrate diets on concentrations of carotenoids, vitamin A and vitamin E in plasma and adipose tissue of lambs. J. Food Compos. Anal..

[bib4] Andonegi A., Garmendia E., Aldezabal A. (2021). Social multi-criteria evaluation for managing biodiversity conservation conflicts. Land Use Policy.

[bib5] Balthazar C.F., Pimentel T.C., Ferrão L.L., Almada C.N., Santillo A., Albenzio M., Mollakhalili N., Mortazavian A.M., Nascimento J.S., Silva M.C., Freitas M.Q., Sant'Ana A.S., Granato D., Cruz A.G. (2017). Sheep milk: Physicochemical characteristics and relevance for functional food development. Compr. Rev. Food Sci. Food Saf..

[bib6] Barron L.J.R., Aldai N., Virto M., De Renobales M., Papademas J., Bintsis T. (2018). Global Cheesemaking Technology: Cheese Quality and Characteristics.

[bib7] Barron L.J.R., Andonegi A., Gamboa G., Garmendia E., García O., Aldai N., Aldezabal A. (2021). Sustainability assessment of pasture-based dairy sheep systems: A multidisciplinary and multiscale approach. Sustainability (Basel).

[bib8] Bernard L., Bonnet M., Delavaud C., Delosière M., Ferlay A., Fougère H., Graulet B. (2018). Milk fat globule in ruminant: Major and minor compounds, nutritional regulation and differences among species. Eur. J. Lipid Sci. Technol..

[bib9] Blanco-Doval A., Barron L.J.R., Bustamante M.A., Aldai N. (2025). Characterization and monitoring of changes during lactation in the profile of multiple bioactive compounds of milk from grazing mares. J. Sci. Food Agric..

[bib10] Cabezuelo M.T., Zaragoza R., Barber T., Viña J.R. (2020). Role of vitamin A in mammary gland development and lactation. Nutrients.

[bib11] Capuano E., van der Veer G., Boerrigter-Eenling R., Elgersma A., Rademaker J., Sterian A., van Ruth S.M. (2014). Verification of fresh grass feeding, pasture grazing and organic farming by cows farm milk fatty acid profile. Food Chem..

[bib12] Cifuni G.F., Claps S., Signorelli F., Di Francia A., Di Napoli M.A. (2022). Fatty acid and terpenoid profile: A signature of mountain milk. Int. Dairy J..

[bib13] Coppa M., Chassaing C., Sibra C., Cornu A., Verbič J., Golecký J., Engel E., Ratel J., Boudon A., Ferlay A., Martin B. (2019). Forage system is the key driver of mountain milk specificity. J. Dairy Sci..

[bib14] de Rancourt M., Fois N., Lavín M.P., Tchakérian E., Vallerand F. (2006). Mediterranean sheep and goats production: An uncertain future. Small Rumin. Res..

[bib15] Debier C., Pottier J., Goffe C., Larondelle Y. (2005). Present knowledge and unexpected behaviours of vitamins A and E in colostrum and milk. Livest. Prod. Sci..

[bib16] Delgado-Pertíñez M., Gutiérrez-Peña R., Mena Y., Fernández-Cabanás V.M., Laberye D. (2013). Milk production, fatty acid composition and vitamin E content of Payoya goats according to grazing level in summer Mediterranean shrublands. Small Rumin. Res..

[bib17] Etaio I., Manso T., Pérez-Elortondo F.J., Gallardo B., Larrasoain L., Ruiz Mantecón A., Lavín P., Barron L.J.R. (2025). Information about production system and attitude toward sustainability: Do they influence the perception in consumers of sheep milk cheese?. J. Dairy Sci..

[bib18] Fantuz F., Salimei E., Papademas P., Tsakalidou E., Papadimitriou K. (2016). Non-Bovine Milk and Milk Products.

[bib19] Fedele V., Rubino R., Claps S., Manzi P., Marconi S., Pizzoferrato M. (2004). Seasonal variation in retinol concentration of goat milk associated with grazing compared to indoor feeding. S. Afr. J. Anim. Sci..

[bib20] Garmendia E., Aldezabal A., Galan E., Andonegi A., Del Prado A., Gamboa G., Garcia O., Pardo G., Aldai N., Barron L.J.R. (2022). Mountain sheep grazing systems provide multiple ecological, socio-economic, and food quality benefits. Agron. Sustain. Dev..

[bib21] Gutiérrez-Peña R., Fernández-Cabanás V.M., Mena Y., Delgado-Pertíñez M. (2018). Fatty acid profile and vitamins A and E contents of milk in goat farms under Mediterranean wood pastures as affected by grazing conditions and seasons. J. Food Compos. Anal..

[bib22] Hulshof P.J.M., van Roekel-Jansen T., van de Bovenkamp P., West C.E. (2006). Variation in retinol and carotenoid content of milk and milk products in the Netherlands. J. Food Compos. Anal..

[bib23] Lomba A., Strohbach M., Jerrentrup S., Dauber J., Klimek S., McCracken D.I. (2017). Making the best of both worlds: Can high-resolution agricultural administrative dates support the assessment of High Nature Value farmlands across Europe?. Ecol. Indic..

[bib24] Lucas A., Agabriel C., Martin B., Ferlay A., Verdier-Metz I., Coulon J., Rock E. (2006). Relationships between the conditions of cow's milk production and the contents of components of nutritional interest in raw milk farmhouse cheese. Lait.

[bib25] Lucas A., Rock E., Chamba J., Verdier-Metz I., Brachet P., Coulon J. (2006). Respective effects of milk composition and the cheese-making process on cheese compositional variability in components of nutritional interest. Lait.

[bib26] Marino V.M., Schadt I. (2016). Stability of α-tocopherol, γ-tocopherol and β-carotene during ripening of pasta-filata cheese made from raw and pasteurised milk with different vitamin contents. Int. Dairy J..

[bib27] Martini M., Salari F., Buttau L., Altomonte I. (2021). Natural content of animal and plant sterols, alpha-tocopherol and fatty acid profile in sheep milk and cheese from mountain farming. Small Rumin. Res..

[bib28] Morand-Fehr P., Fedele V., Decandia M., Le Frileux Y. (2007). Influence of farming and feeding systems on composition and quality of goat and sheep milk. Small Rumin. Res..

[bib29] Moscovici Joubran A., Pierce K.M., Garvey N., Shalloo L., O'Callaghan T.F. (2021). Invited review: A 2020 perspective on pasture-based dairy systems and products. J. Dairy Sci..

[bib30] Nájera A.I., Bustamante M.A., Albisu M., Valdivielso I., Amores G., Mandaluniz N., Arranz J., Barron L.J.R., De Renobales M. (2017). Fatty acids, vitamins and cholesterol content, and sensory properties of cheese made with milk from sheep fed rapeseed oilcake. J. Dairy Sci..

[bib31] Nozière P., Grolier P., Durand D., Ferlay A., Pradel P., Martin B. (2006). Variations in carotenoids, fat-soluble micronutrients, and color in cows 'plasma and milk following changes in forage and feeding levels. J. Dairy Sci..

[bib32] O'Callaghan T.F., Mannion D.T., Hennessy D., McAuliffe S., O'Sullivan M.G., Leeuwendaal N., Beresford T.P., Dillon P., Kilcawley K.N., Sheehan J.J., Ross R.P., Stanton C. (2017). Effect of pasture versus indoor feeding systems on quality characteristics, nutritional composition and sensory and volatile properties of full-fat Cheddar cheese. J. Dairy Sci..

[bib33] Panfili G., Manzi P., Pizzoferrato L. (1994). High-performance liquid chromatographic method for the simultaneous determination of tocopherols, carotenes, and retinol and its geometric isomers in Italian cheeses. Analyst.

[bib34] Pizzoferrato L., Manzi P., Marconi S., Fedele V., Claps S., Rubino R. (2007). Degree of antioxidant protection: a parameter to trace the origin and quality of goat's milk and cheese. J. Dairy Sci..

[bib35] Raynal-Ljutovac K., Lagriffoul G., Paccard P., Guillet I., Chilliard Y. (2008). Composition of goat and sheep milk products: An update. Small Rumin. Res..

[bib36] Rocchi S., Caretti F., Gentili A., Curini R., Perret D., Pérez-Fernández V. (2016). Quantitative profiling of retinyl esters in milk from different ruminant species by using high performance liquid chromatography-diode array detection-tandem mass spectrometry. Food Chem..

[bib37] Valdivielso I., Bustamante M.A., Aldezabal A., Amores G., Virto M., Ruiz De Gordoa J.C., De Renobales M., Barron L.J.R. (2016). Case study of a commercial sheep flock under extensive mountain grazing: Pasture derived lipid compounds in milk and cheese. Food Chem..

[bib38] Valdivielso I., Bustamante M.A., Buccioni A., Franci O., Ruiz De Gordoa J.C., De Renobales M., Barron L.J.R. (2015). Commercial sheep flocks—Fatty acid and fat-soluble antioxidant composition of milk and cheese related to changes in feeding management throughout lactation. J. Dairy Res..

